# Antiretroviral Long-Term Efficacy and Resistance of Lopinavir/Ritonavir Plus Lamivudine in HIV-1-Infected Treatment-Naïve Patients (ALTERLL): 144-Week Results of a Randomized, Open-Label, Non-Inferiority Study From Guangdong, China

**DOI:** 10.3389/fphar.2020.569766

**Published:** 2021-03-25

**Authors:** Peng-Le Guo, Hao-Lan He, Xie-Jie Chen, Jin-Feng Chen, Xiao-Ting Chen, Yun Lan, Jian Wang, Pei-Shan Du, Huo-Lin Zhong, Hong Li, Cong Liu, Li-Ya Li, Feng-Yu Hu, Xiao-Ping Tang, Wei-Ping Cai, Ling-Hua Li

**Affiliations:** Guangzhou Eighth People’s Hospital, Guangzhou Medical University, Guangzhou, China

**Keywords:** antiretroviral therapy, simplified regimen, randomized controlled study, lopinavir/ritonavir, inflammatory biomarker, efavirenz

## Abstract

Dual therapy with lopinavir/ritonavir (LPV/r) plus lamivudine (3TC) has been demonstrated to be non-inferior to the triple drug regimen including LPV/r plus two nucleoside reverse transcriptase inhibitors (NRTIs) in 48-week studies. However, little is known about the long-term efficacy and drug resistance of this simplified strategy. A randomized, controlled, open-label, non-inferiority trial (ALTERLL) was conducted to assess the efficacy, drug resistance, and safety of dual therapy with LPV/r plus 3TC (DT group), compared with the first-line triple-therapy regimen containing tenofovir (TDF), 3TC plus efavirenz (EFV) (TT group) in antiretroviral therapy (ART)-naïve HIV-1–infected adults in Guangdong, China. The primary endpoint was the proportion of patients with plasma HIV-1 RNA < 50 copies/ml at week 144. Between March 1 and December 31, 2015, a total of 196 patients (from 274 patients screened) were included and randomly assigned to either the DT group (n = 99) or the TT group (n = 97). In the primary intention-to-treat (ITT) analysis at week 144, 95 patients (96%) in the DT group and 93 patients (95.9%) in the TT group achieved virological inhibition with plasma HIV-1 RNA <50 copies/ml (difference: 0.1%; 95% CI, –4.6–4.7%), meeting the criteria for non-inferiority. The DT group did not show significant differences in the mean increase in CD4^+^ cell count (247.0 vs. 204.5 cells/mm^3^; *p* = 0.074) or the CD4/CD8 ratio (0.47 vs. 0.49; *p* = 0.947) from baseline, or the inflammatory biomarker levels through week 144 compared with the TT group. For the subgroup analysis, baseline high viremia (HIV-1 RNA > 100,000 copies/ml) and genotype BC did not affect the primary endpoint or the mean increase in CD4^+^ cell count or CD4/CD8 ratio from baseline at week 144. However, in patients with genotype AE, the DT group showed a higher mean increase in CD4^+^ cell count from baseline through 144 weeks than the TT group (308.7 vs. 209.4 cells/mm^3^; *p* = 0.038). No secondary HIV resistance was observed in either group. Moreover, no severe adverse event (SAE) or death was observed in any group. Nonetheless, more patients in the TT group (6.1%) discontinued the assigned regimen than those in the DT group (1%) due to adverse events. Dual therapy with LPV/r plus 3TC manifests long-term non-inferior therapeutic efficacy, low drug resistance, good safety, and tolerability compared with the first-line triple-therapy regimen in Guangdong, China, indicating dual therapy is a viable alternative in resource-limited areas.

**Clinical Trial Registration:** [http://www.chictr.org.cn], identifier [ChiCTR1900024611].

## Introduction

Combined antiretroviral therapy (ART) for the treatment of HIV infection was established nearly 30 years ago, significantly decreasing AIDS-related morbidity and mortality globally (Gulick et al., 1997; Gunthard et al., 2016). In general, ART combination therapy comprises a three-drug regimen (Gunthard et al., 2016). In China, the government currently provides free ART for Chinese HIV-infected patients, with treatment comprising a first-line regimen of tenofovir disoproxil fumarate (TDF) plus lamivudine (3TC) plus efavirenz (EFV), and a second-line regimen of lopinavir/ritonavir (LPV/r) plus two nucleoside reverse transcriptase inhibitors (NRTIs) (Ning et al., 2017). However, in patients with a contraindication for TDF and could not obtain other NRTI alternatives, TDF-free simplified therapy, a potentially important alternative, holds several advantages, including low drug toxicity, lower pill burden, better adherence, and increased drug reserves in the future (Ahamed et al., 2016; Chwiki et al., 2017; De La Mata et al., 2017).

In recent years, simplified treatment strategies have garnered considerable interest. The first simplified strategy evaluated involved a boosted protease inhibitor (bPI) with a high genetic barrier to resistance. To date, dual therapy (DT) with bPI and 3TC has been found to be non-inferior to triple ART in multiple clinical trials (Calza et al., 2018; Perez-Molina et al., 2015), and is recognized as a confirmed switch therapy in current HIV treatment guidelines for use in special situations (European AIDS Clinical Society, 2017). In addition, in two separate studies, a simplified treatment regimen comprising LPV/r plus 3TC was demonstrated to be non-inferior to the standard treatment regimen in both antiretroviral therapy–naive and virologically suppressed HIV patients at week 48 (Cahn et al., 2014; Arribas et al., 2015).

However, a comprehensive comparison of EFV-based triple therapy (TT) vs. simplified strategies in HIV patients in China has not been published. To address this knowledge gap, we conducted a randomized, controlled, open-label, non-inferiority trial in antiretroviral-naïve HIV-1–infected patients comparing LPV/r plus 3TC DT with the Chinese first-line triple-therapy regimen (TDF, 3TC plus EFV). In China, where LPV/r is the only free protease inhibitor available to HIV patients, LPV/r is widely used (as it is in other countries with limited resources). We previously reported that DT with LPV/r plus 3TC was non-inferior to the first-line triple-therapy regimen at week 48 (Li et al., 2018). The study (ALTERLL) was continued using the same cohort, and we report here the antiretroviral long-term efficacy and safety of these regimens up to 144 weeks.

## Materials and Methods

### Study Design

This study reports the results of a randomized, controlled, open-label, non-inferiority trial started in March 2015 comparing antiretroviral long-term efficacy and resistance in a DT regimen of LPV/r plus 3TC vs. a triple-therapy regimen of TDF plus 3TC plus EFV. Our research was conducted on the hypothesis of no difference between the two groups. Patients were eligible for enrollment if they were infected with HIV-1, older than 18 years of age, naive to ART, and had a CD4^+^ cell count over 200 cells/mm^3^ at the time of screening. The exclusion criteria included a) pregnancy or breastfeeding, b) coinfected with HBV or HCV, and c) complications from chronic liver disease or AIDS-associated opportunistic diseases within 30 days before screening.

Our research was divided into two phases, up to 48 weeks and 48–144 weeks. The results obtained at 48 weeks have previously been published (Li et al., 2018). Here, the time window for this study was extended to 144 weeks. The study was conducted at Guangzhou Eighth People’s Hospital, which is an infectious disease specialist hospital following up around 15,000 HIV-infected patients receiving long-term ART. The study was approved by the Ethics Committee of the Guangzhou Eighth People’s Hospital (Approval No. 20142154). In addition, written informed consent was obtained from all participants. This study was registered with the Chinese Clinical Trial Registry, number ChiCTR1900024611.

### Randomization and Masking

A high viral load (usually considered HIV-1 RNA >100,000 copies/ml) may affect the efficacy of ART. To reduce the influence of this confounding factor, a stratified analysis was performed according to the screening level of plasma HIV-1 RNA (≤100,000 vs. >100,000 copies/ml). In each of the two stratifications, patients were randomly divided into two groups (at a ratio of 1:1) according to a computer-generated allocation schedule, and patients in each group received DT or TT. DT included lopinavir 400 mg and ritonavir 100 mg twice daily plus 3TC 300 mg once daily. TT consisted of TDF 300 mg plus 3TC 300 mg plus EFV 600 mg, all once daily. Since the study was open label, patients and investigators were unmasked to treatment allocation.

### Procedures

Each patient attending the clinic was screened according to the inclusion and exclusion criteria. In total, 274 patients were screened, and 196 patients were eligible for inclusion and randomly assigned to either DT (n = 99) or TT (n = 97). Patients were assessed on the screening day, day 1 (baseline), and at weeks 12, 24, 48, 72, 96, 120, and 144, or at early termination. Plasma HIV-1 RNA was quantified, and blood specimens were preserved at baseline, weeks 12, 24, 48, 96, and 144. Clinical assessments were performed at every visit. HIV genotypic assays were performed at screening. Resistance testing was performed at screening and upon the development of confirmed protocol-defined virological failure. Virological failure was defined as two consecutive viral loads (≥7 days and ≤30 days apart) of more than 400 copies/ml at week 24 or later after ART.

All laboratory data were obtained from the central laboratory of the Guangzhou Eighth People’s Hospital. This laboratory met all the requirements of the Clinical Laboratory Improvement Amendments regulations and the National Guideline for Detection of HIV/AIDS (Society of Infectious Diseases, 2015). The estimated glomerular filtration rate (eGFR) was calculated using the 2009CKD-EPI equation, and reduced eGFR was defined as an eGFR <80 ml/min/1.73 m^2^ or a reduction in the GFR of 10% in the last year (Inker et al., 2012). The chronic immune activation indicators utilized in this study included CD4^+^CD38^+^, CD8^+^CD38^+^, CD4^+^HLA-DR^+^, CD8^+^HLA-DR^+^, CD4^+^CD69^+^, and CD8^+^CD69^+^ cells.

Adverse events, including discomfort symptoms, signs, and abnormal laboratory examinations, were compiled from baseline through 144 weeks.

### Subgenotype Determination and Drug Resistance Gene Mutation Analysis

Plasma HIV-1 RNA load was detected using the Roche COBAS-TaqMan Assay (HIV-1 Test, version 2.0, Indianapolis, IN, United states). The target fragment for the subgenotyping includes 99 amino acid coding codons from the protease (PR) region, and resistance analysis consists of approximately 300 amino acid coding codons from the reverse transcriptase region (primer sequences listed in Supplementary Table S1). The subgenotype of HIV-1 was tested using Bioedit and MEGA5 software with the standard strains selected from the Los Alamos HIV database, and mutation sites were identified using the Stanford HIV-resistant database (Rhee et al., 2017; Zhang et al., 2017).

### Flow Cytometric Analysis of T Cell Activation Indicators

Whole blood samples (5 ml) were collected from patients, and their peripheral blood mononuclear cells were isolated and preserved. The samples were cryopreserved at −80°C in 80% fetal calf serum, 10% RPMI–1640, and 10% dimethyl sulfoxide (DMSO) (Sigma-Aldrich, St. Louis, MO, LT: 67–68–5). The following human monoclonal antibodies were used for labeling: antihuman CD4-APC-H7 (Cat: 580158, LT: 7062573), antihuman CD8-FITC (Cat: 555634, LT: 6195899), antihuman HLA-DR-APC (Cat: 559866, LT: 6014674), antihuman CD69-BV421 (Cat: 562884, LT: 6154722), and antihuman CD38-PR-Cy^TM^7 (Cat: 561646, LT: 7163804). These antibodies were purchased from BD Biosciences (San Jose, CA, United States).

### Outcomes

The primary endpoint was the proportion of subjects with plasma HIV-1 RNA loads <50 copies/ml at week 144, referred to as virological response rate (Ning et al., 2017). Secondary endpoints included the change in CD4^+^ cell count and ratio of CD4^+^ and CD8^+^, resistance profile, adverse events, and changes in chronic immune activation indicators. If patients in the DT group developed virological failure, they were switched to receive TT on the basis of the national free ART directory (De La Mata et al., 2017; Ning et al., 2017).

### Statistical Analyses

Estimation of optimal sample size was based on the assumption of no difference between the two groups and a predicted efficacy of 85% at week 144. In the ITT analysis with a 15% margin and a 10% withdrawal rate, we calculated that 99 participants per arm would be required.

Efficacy was analyzed in the ITT population. All randomized participants were included in the analysis based on the intentionality principle. Per-protocol (PP) population refers to all participants who completed the 144-week treatment and had no major protocol violations. Safety was analyzed in the safety population, defined as all subjects who received at least one dose of study medications. Subgroup analysis was carried out to analyze outcomes in patients with different baseline viral loads and genotypes.

All statistical analyses were performed using IBM SPSS Statistics 21 and MedCalc 16 software. Quantitative data are reported as mean ± standard deviation (SD), while qualitative data are reported as numbers and proportions. The Mann–Whitney *U* test was used to compare two groups of quantitative or ordinal data, and Fisher’s exact test was used to compare the nominal data. For all statistical data, a double-tailed test with a level of 0.05 was used, and differences with a *p*-value of less than 0.05 were considered significant.

## Results

### Basic Data of Patients

**FIGURE 1:**
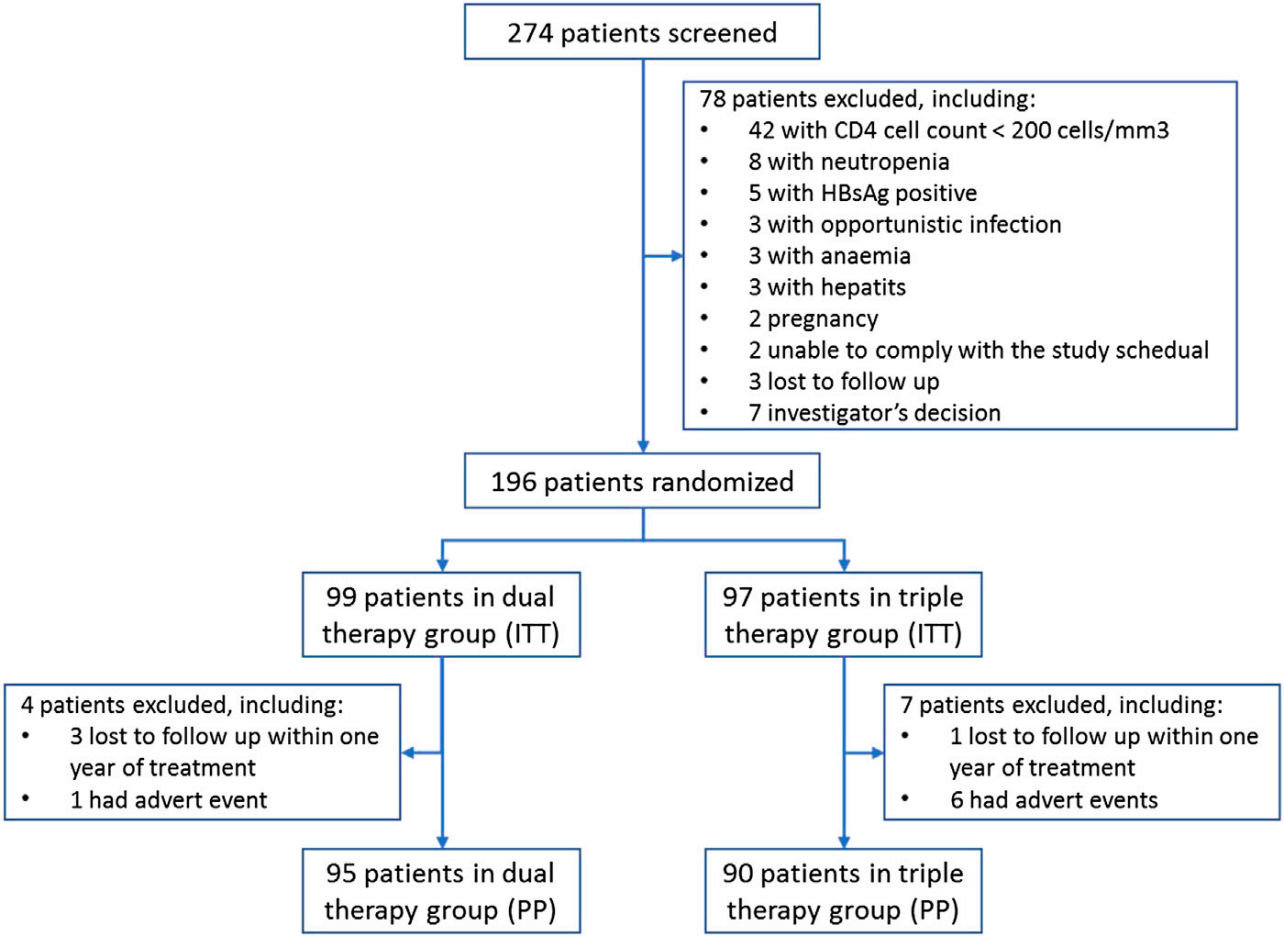
Subject disposition of the study.

A summary of subject characteristics is presented in Figure 1. Seven patients in the TT group and four patients in the DT group were excluded from the PP population due to loss during follow-up (n = 4) or adverse events (n = 7). No significant differences in the demographics and baseline characteristics of the ITT population were observed between the treatment groups (Table 1).

**TABLE 1:** Demographics and baseline characteristics (intention-to-treat population).

	Dual therapy (n = 99)	Triple therapy (n = 97)	*p*-Value
**Demographics**			
Age, median (IQR), years	28 (25, 33)	29 (25, 36)	0.265
Male, n (%)	95 (96.0)	88 (90.7)	0.141
Female, n (%)	4 (4.0%)	9 (9.3%)	0.206
BMI, median (IQR), kg/m^2^	20.7 (18.9, 23.2)	20.8 (19.3, 23.7)	0.668
**Baseline disease characteristics**			
HIV RNA, median (IQR), log 10 copies/mL	4.4 (3.8, 4.7)	4.4 (4.0, 4.7)	0.668
HIV RNA, copies/mL, n (%)			0.290
≤100,000	85 (85.9)	88 (90.7)	
>100,000	14 (14.1)	9 (9.3)	
CD4^+^ cell count, median (IQR), cells/mm^3^	330 (263, 400)	327 (264, 384)	0.626
CD4^+^ cell count, cells/mm^3^, n (%)			0.904
>200 ≤ 350	58 (58.6)	56 (57.7)	
>350	41 (41.4)	41 (42.3)	
Ratio of CD4 and CD8, median (IQR)	0.33 (0.24, 0.45)	0.30 (0.23, 0.41)	0.268
HIV-1 subtype, n (%)			0.516
CRF01_AE	28 (28.3)	28 (28.9)	
CRF07_BC	46 (46.5)	48 (49.5)	
CRF08_BC	4 (4.0)	5 (5.2)	
CRF55_01B	12 (12.1)	5 (5.2)	
CRF58_01B	1 (1.0)	1 (1.0)	
CRF59_01B	2 (2.0)	2 (2.1)	
B	2 (2.0)	6 (6.2)	
Other	2 (2.0)	4 (4.1)	

### Efficacy Outcomes

**FIGURE 2:**
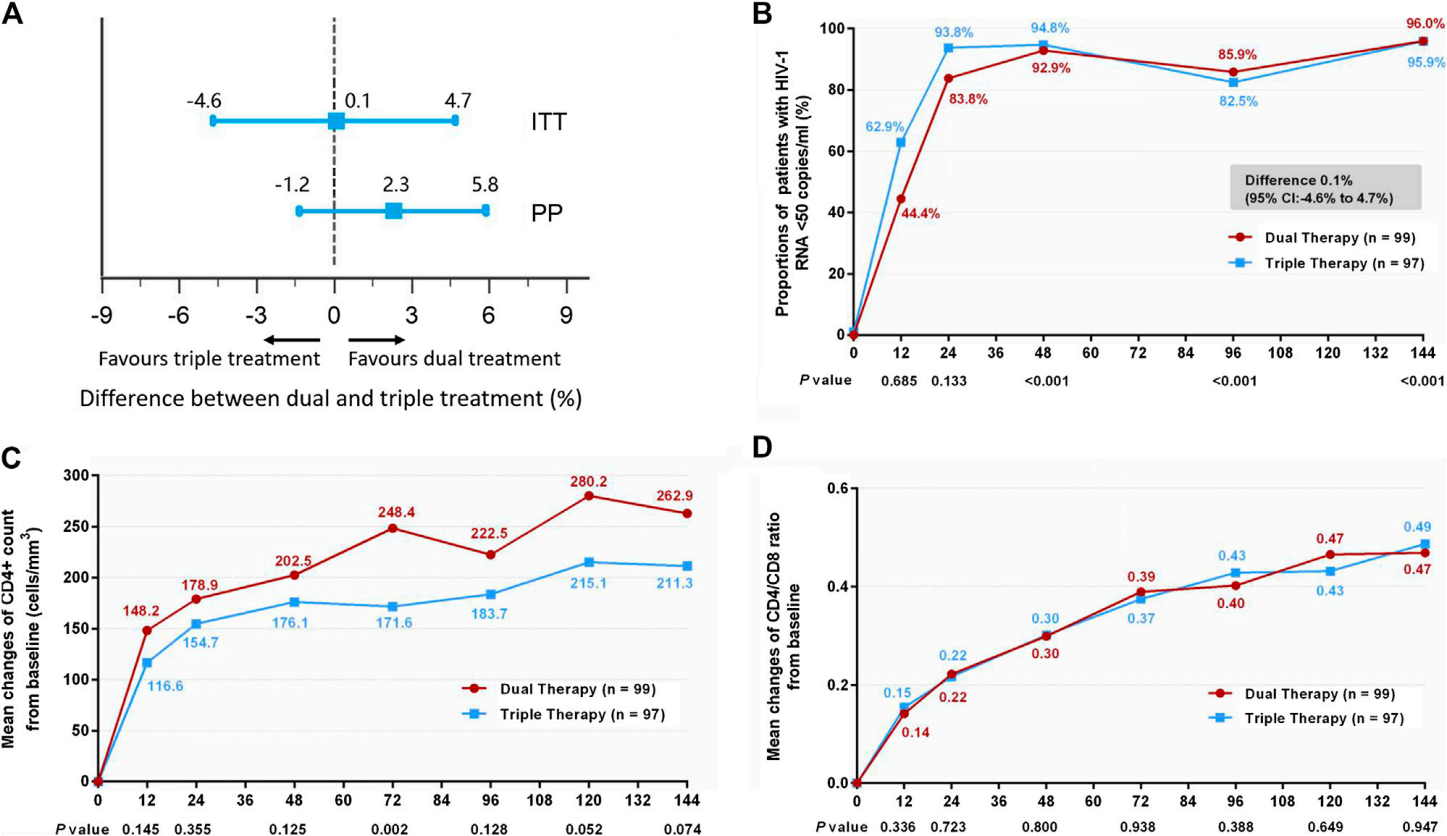
**(A)** Therapeutic response in the ITT and PP populations. The solid line represents no difference (dual treatment minus triple treatment) and the 15% non-inferiority margin. **(B)** Proportion of patients with HIV-1 RNA <50 copies/ml in ITT analysis. Proportions from two groups at different time points were tested for non-inferiority. *p* < 0.05 indicates that the dual treatment group is non-inferior to the triple treatment group. **(C)** Change in CD4^+^ count from baseline in ITT analysis. CD4^+^ counts from the two groups at different time points were tested with a Mann–Whitney *U* test. A *p*-value of less than 0.05 was considered significant. **(D)** Change in CD4/CD8 ratio from baseline in ITT analysis. CD4/CD8 ratios from the two groups at different time points were tested with a Mann–Whitney *U* test. A *p*-value less than 0.05 was considered significant.

The proportion of patients in the DT group who reached the primary efficacy endpoint was non-inferior to that achieved in the TT group (Figure 2A). Thus, 95 patients (96.0%) in the DT group and 93 patients (95.9%) in the TT group demonstrated HIV-1 RNA loads of <50 copies/ml at week 144 (difference 0.1%: 95% CI, −4.6–4.7%) in the ITT analysis (Figure 2B). In the PP analysis, the proportions were 98.9% (94/95) in the DT group and 96.7% (87/90) in the TT group (difference 2.3%: 95% CI, −1.2–5.8%; Supplementary Figure S2B).

Although the DT group showed higher increases in CD4^+^ cell count from baseline through 144 weeks than the TT group, both in the ITT (Figure 2C) (median, 247.0 cells/mm^3^ in DT group vs. 204.5 cells/mm^3^ in TT group) and PP (Supplementary Figure S2C) (median, 251.5 cells/mm^3^ in DT group vs. 204.5 cells/mm^3^ in TT group) analyses, the differences were not statistically significant (*p* = 0.074 and *p* = 0.075, respectively). A similar increase in the ratio of CD4^+^ to CD8^+^ was observed in the two groups, both in the ITT (Figure 2D) (median, 0.45 in DT group vs. 0.46 in TT group) and PP (Supplementary Figure S2D) (median, 0.45 in DT group vs. 0.45 in TT group) analyses.

**FIGURE 3:**
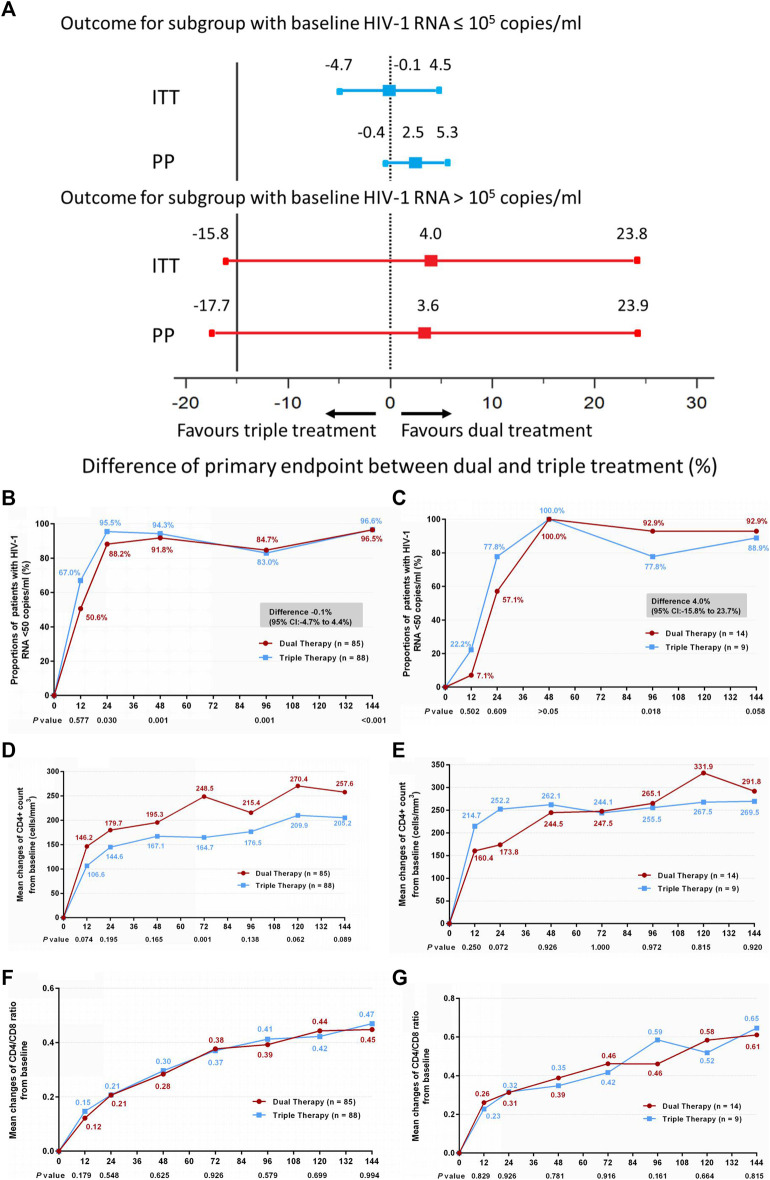
**(A)** Therapeutic response in the ITT and PP populations in subgroups stratified by baseline HIV-1 RNA. Solid lines represent no difference (dual treatment minus triple treatment) and the 15% non-inferiority margin **(B,C)**. **(B)** Proportion of patients with HIV-1 RNA <50 copies/ml; proportion of patients with a baseline HIV-1 RNA ≤100,000 copies/ml in ITT analysis. **(C)** proportion of patients with a baseline HIV-1 RNA >100,000 copies/ml in ITT analysis. Proportions obtained from two groups at different time points were tested for non-inferiority. A *p* < 0.05 indicates that dual treatment group is non-inferior to the triple treatment group **(D,E)**. **(D)** Change in CD4^+^ count from baseline in patients with a baseline HIV-1 RNA ≤100,000 copies/ml in ITT analysis. **(E)** Change in CD4^+^ count from baseline in patients with a baseline HIV-1 RNA >100,000 copies/ml in ITT analysis. CD4^+^ counts from the two groups at different time points were tested with a Mann–Whitney *U* test. A *p*-value of less than 0.05 was considered significant **(F,G)**. **(F)** Change in CD4/CD8 ratio from baseline in patients with a baseline HIV-1 RNA ≤100,000 copies/mL in ITT analysis. **(G)** Change in CD4/CD8 ratio from baseline in patients with a baseline HIV-1 RNA >100,000 copies/mL in ITT analysis. CD4/CD8 ratio from the two groups at different time points were tested with a Mann–Whitney *U* test. A *p*-value of less than 0.05 was considered significant.

Subgroup analysis was also conducted according to baseline HIV-1 RNA load. Within each subgroup (baseline HIV-1 RNA ≤ 100,000 copies/ml and baseline HIV-1 RNA > 100,000 copies/ml), no significant differences in the primary endpoint were observed between the DT and TT groups (Figures 3A–C) (difference −0.1%: 95% CI, −4.7–4.4% for the baseline HIV-1 RNA ≤100,000 copies/ml subgroup; difference 4.0%: 95% CI, −15.8–23.7% for the baseline HIV-1 RNA >100,000 copies/ml subgroup). Moreover, no significant difference was observed in the increase in CD4^+^ cell count from baseline through 144 weeks (Figures 3D,E) (median, 247 cells/mm^3^ vs. 190 cells/mm^3^ and 267.5 cells/mm^3^ vs. 264 cells/mm^3^ in the low load and high load subgroup, respectively), or in the ratio increase of CD4 to CD8 (Figures 3F,G) (median, 0.44 vs. 0.45 and 0.58 vs. 0.67 in the low load and high load subgroup, respectively). The results of PP analysis are consistent with those of the ITT analysis (Supplementary Figures S3B–G).

**FIGURE 4:**
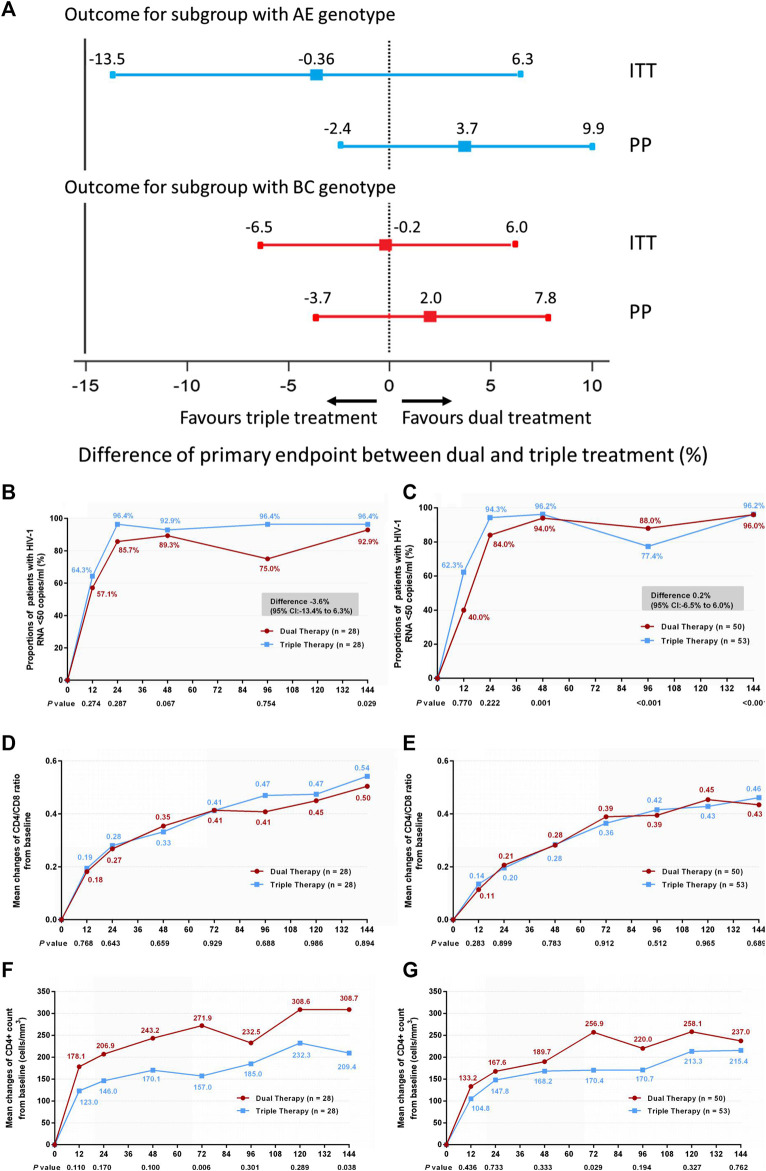
**(A)** Therapeutic response in the ITT and PP populations in subgroups stratified according to genotype. Solid lines represent no difference (dual treatment minus triple treatment) and the 15% non-inferiority margin **(B,C)**. **(B)** Proportion of patients with HIV-1 RNA <50 copies/ml and with AE genotype in ITT analysis. **(C)** Proportion of patients with HIV-1 RNA <50 copies/ml and with BC genotype in ITT analysis. Proportions from two groups at different time points were tested for non-inferiority. A *p* < 0.05 indicates that dual treatment group is non-inferior to the triple treatment group **(D,E)**. **(D)** Change in CD4/CD8 ratio from baseline in patients with AE genotype in ITT analysis. **(E)** Change in CD4/CD8 ratio from baseline in patients BC genotype in ITT analysis. CD4^+^ counts from the two groups at different time points were tested with a Mann–Whitney *U* test. A *p*-value of less than 0.05 was considered significant **(F,G)**. **(F)** Change in CD4^+^ count from baseline in patients with AE genotype in ITT analysis. **(G)** Change in CD4^+^ count from baseline in patients with BC genotype in ITT analysis. CD4/CD8 ratios from the two groups at different time points were tested with a Mann–Whitney *U* test. A *p*-value of less than 0.05 was considered significant.

In addition, we compared treatment efficacy between the two treatment groups in patients with either genotype AE or genotype BC at week 144. No significant difference was observed in the proportions of patients with HIV-1 RNA loads <50 copies/ml **(** Figures 4A–C) (difference −3.6%: 95% CI, −13.4–6.3% in patients with genotype AE; difference 0.2%: 95% CI, −6.5–6.0% in patients with genotype BC), or in the increases in CD4/CD8 ratio (Figures 4D,E) (0.53 in both DT and TT groups, z = 0.134, *p* = 0.894). In patients with the AE genotype, the DT group showed a greater increase in CD4^+^ cell count from baseline through 144 weeks than the TT group (median, 289 cells/mm^3^ in the DT group vs. 173 cells/mm^3^ in the TT group, *z* = 2.076, *p* = 0.038). No significant difference in the elevation of CD4^+^ cell count was observed between the subgroups in patients with the BC genotype (median, 219.3 cells/mm^3^ in the DT group vs. 231.5 cells/mm^3^ in the TT group) (Figures 4F,G). The results of PP analysis were consistent with those of the ITT analysis (Supplementary Figures S4B–G).

The chronic immune activation indicators, including CD4^+^CD38^+^, CD8^+^CD38^+^, CD4^+^HLA-DR^+^, CD8^+^HLA-DR^+^, CD4^+^CD69^+^, and CD8^+^CD69^+^ cells, were measured from baseline through 144 weeks. In both treatment groups, CD4^+^CD38^+^ and CD4^+^CD69^+^ increased in the first year after treatment, and then decreased over the following 2 years. CD4^+^HLA-DR^+^ showed a slight elevation trend during the 144 weeks, while CD8^+^CD38^+^, CD8^+^HLA-DR^+^, and CD8^+^CD69^+^ generally exhibited a downward trend from baseline through 144 weeks. However, no significant difference in the overall trend between the two groups over 144 weeks was observed (Supplementary Figure S5).

### Resistance Profile

Only two participants in the DT arm (2%) and two participants in the TT arm (2.06%) developed protocol-defined virological failure (difference, 0.04%: 95% CI −5.31–5.57%). Of these four patients, three decided to discontinue ART (two in the DT group and one in the TT group). The quantitative plasma viral load of the remaining patient was 401 copies/ml.

The primary drug resistance results of the 196 patients were as follows: 28 resistant mutations were detected; six cases showed altered sensitivities to the drugs (a drug resistance rate of 3.06%). Of the six cases of altered sensitivity, one case (in the DT group) was mildly resistant to PI, two cases (in the TT group) were resistant to NRTIs, and three cases (in the DT group) were resistant to non-NRTIs (NNRTIs); no cross-resistance was observed. None of the amplified samples at treatment failure in either group showed any secondary resistance mutations. No mutations associated with protease inhibitors were identified in either arm.

### Safety Outcomes

A majority of the patients experienced at least one adverse event during the 144-week study period. In total, 195 patients reported adverse events, including 99 in the DT group and 96 in the TT group. More diarrhea cases were reported in the DT group (52.5% in DT group vs. 5.2% in TT group, *p* < 0.001), and more cases of rash, dizziness, insomnia, and “dreaminess” were reported in the TT group (8.1%, 3%, 3%, and 1% in the DT group vs. 23.7%, 53.6%, 15.5%, and 17.5% in TT group, respectively; *p* < 0.05). Regarding laboratory abnormalities, triglyceride and cholesterol elevations were higher in the DT group than in the TT group (18.2% and 8.1% vs. 5.2% and 2.1%, respectively).

More patients (n = 6, 6.1%) in the TT group discontinued the assigned treatment regimen due to adverse events than those in the DT group (n = 1, 1%). In this study, diarrhea caused by LPV/r was common (52.5%); all cases were classified as grade 1–2 diarrhea, and cases were mostly reported in the first month of treatment. The majority of these patients found relief from diarrhea without recourse to prescribed treatment; in a few cases, treatment with montmorillonite powder for a short period was prescribed. None of the patients changed their treatment schedule due to intolerable diarrhea.

Among level 3–4 abnormal laboratory indexes, one case of alanine transaminase (ALT) elevation and two cases of aspartate aminotransferase elevation were reported; all cases were classified as level 3, and a return to normal levels was observed after a 4-week treatment with oral hepato-protective agents. In addition, there were 10 cases with elevated total cholesterol (CHOL) in the DT group, 23 cases with elevated triglyceride, and 10 cases with elevated low-density lipoprotein; all were grade 3–4 abnormalities. However, all abnormal indexes in blood lipid levels improved after application of oral lipid-lowering agents. No hospitalization or life-threatening consequences were found in either group. No severe adverse event (SAE) or death was observed in either group (see data in Supplementary Table S2).

## Discussion

Three-drug combination ART has become available and widely accepted since 1996, dramatically improving the prognosis of people living with HIV (Odone et al., 2014; Sezgin et al., 2018). However, ART is a lifelong commitment, and is associated with several significant problems, including long-term toxicities, potential drug–drug interactions, and a high pill burden, which ultimately lead to a reduction in drug adherence, resulting in frequent treatment discontinuations and the development of drug resistance (Blasco et al., 2013; Tourret et al., 2013; Moyle et al., 2015; Gatell Artigas et al., 2016). Due to the increased viral potency of modern antiretroviral drugs, repositioning to a two-drug combination therapy should be considered, potentially reducing adverse events, drug–drug interactions, and cost, while maintaining an excellent sustained antiviral effect. Various combinations of DT regimens have been studied. The combination of 3TC and a protease inhibitor with booster has shown non-inferiority in several randomized studies (Cahn et al., 2014; Perez-Molina et al., 2015).

In this ALTERLL study, we demonstrate long-term sustained virologic efficacy of DT with lamivudine plus LPV/r over 144 weeks (3 years), with only rare cases developing virologic failure (2%, 2/99). DT with LPV/r plus 3TC is not inferior to the first-line regimen containing TDF, 3TC plus EFV, which is consistent with other findings (Cahn et al., 2014). The characteristics of LPV/r include a high gene barrier and high curative effect, which theoretically guarantees the efficacy of DT with lamivudine plus LPV/r. In practice, the GARDEL study (Cahn et al., 2014) and this study both confirm that DT with lamivudine plus LPV/r is not inferior to TT. In our study, DT was compared with 2NRTIS plus EFV, whereas in the GARDEL study, DT was compared with 2NRTIS plus LPV/r.

In comparison with the results obtained at 48 weeks (Li et al., 2018), the long-term effectiveness reported here at 144 weeks is more convincing. To confirm that dual ART is still effective in high HIV viral load populations and in different HIV genotypes, subgroup analyses according to baseline HIV-1 RNA and genotype were conducted. No significant differences were observed in the viral suppression rates at week 144 between the two groups among patients with high baseline viremia (HIV-1 RNA >100,000 copies/ml) or among patients with different genotypes. However, it should be noted that the number of cases with HIV-1 RNA >100,000 copies/ml was small, resulting in the 95% CI of the rate difference being too wide, and the lower limit exceeding −15%, which might affect the statistical results.

Regarding the chronic immune index, which reflects the restoration of immunity, no significant differences were observed in the increase in the ratio of CD4^+^ to CD8^+^ cells at week 144 between the two groups (or in the relevant subgroups with high/low baseline viremia or different genotypes). However, the DT group demonstrated a greater improvement in CD4^+^ cell count from baseline through 144 weeks than the TT group (although the differences were not statistically significant) and among patients with genotype AE (*p* < 0.05). Thus, it can be inferred from this study that simplified treatment of LPV/r plus 3TC is non-inferior to the standard triple regimen including TDF and 3TC plus EFV, and that DT may have advantages in some specific cases.

Another important concern is that of the improvement of chronic immune activation, for example, inflammatory cytokines (TNFα, IL-6), monocyte activation factor (soluble CD14), T-cell activation factors (HLA-DR, CD38), and exhaustion factor (PD-1) (Bastard et al., 2012; Hatano et al., 2013; Mendez-Lagares et al., 2013; Falasca et al., 2017). These immunity indicators have been reported to be associated with viral escape of HIV replication in sanctuaries, such as the brain and lymph nodes, where drugs may not reach the appropriate concentrations (Huang et al., 2016; Estes et al., 2017; Fletcher et al., 2018). In our study, there was a tendency for the T-cell activation markers to return to normal levels over 144 weeks in both groups, and no significant difference was observed between the two groups. This observation explains why DT is not inferior to TT in terms of virus suppression rate and immune reconstitution, and why DT can maintain a long-term effect over 144 weeks.

Virological failure was very rare in both arms of the study. Only four participants had viral rebounds >400 HIV-RNA copies/mL; treatment in three of these cases was subsequently discontinued due to low compliance. The quantitative plasma viral load of the remaining case was 401 copies/ml, which was lower than the viral load standard for drug resistance. In fact, in some similar simplified treatment studies, the presence of resistant sites, even for the NRTIs with low resistance barriers, cannot be induced. For example, in the OLE study, the observed drug resistance was due to previous irregular exposure to lamivudine (Arribas et al., 2015). In a simplified treatment study containing lamivudine and darunavir/ritonavir, virus rebound did not reveal a resistance site for lamivudine (Pulido et al., 2017). In the present study, no secondary resistance sites were observed, even if drug resistance was detected before treatment.

Thus far, current research has not reached a consensus on the adverse events associated with simplified treatment of LPV/r plus 3TC. In the SWORD one and two studies, adverse events were reported to be more frequent, and the incidence of discontinuation was increased in the arm receiving simplified treatment with LPV/r (Llibre et al., 2018). In the GEMINI study, significant differences in renal markers and bone markers were reported in the arms receiving DT or TT containing LPV/r at week 48 (Cahn et al., 2019). In our study, more patients in the TT group discontinued the assigned treatment regimen due to adverse events than those in the DT group, although the schedule containing lopinavir/ritonavir had higher incidences of diarrhea and metabolic disorders. Hyperlipidemia caused by LPV/r has been widely reported (Dai et al., 2019; Huang et al., 2019; Su et al., 2019). In agreement with these observations, our results confirm the occurrence of hyperlipidemia in the DT group, and show its side effects and its relation to other factors. For example, a rise in blood lipid levels in HIV-infected patients was more common than expected than in the general population (Grunfeld, 2010; Jain et al., 2018; Waters and Hsue, 2019). It should be noted that some patients had dyslipidemia before treatment, and that the extended follow-up time for this study increased the overall probability of dyslipidemia.

Our ALTERLL study is not without limitations. The limitations include the exclusion of CD4 < 200 patients, and the single-center, non–double-blind design of our study. However, we were still able to confirm that DT with LPV/r plus 3TC can maintain good long-term virologic efficacy. Furthermore, immune reconstitution in ART-naïve HIV-1–infected patients with genotype AE is greater following DT than immune reconstitution in patients following TT therapy with the first-line regimen TDF, 3TC plus EFV. In addition, drug resistance was relatively low in patients who had received DT (even after 144 weeks of treatment). Although LPV/r may lead to more metabolic disorders, these adverse events appear to be endurable and controllable. In conclusion, DT with LPV/r plus 3TC is a viable alternative in ART-naïve HIV-1–infected patients in resource-limited areas, including China.

## Data Availability

The raw data supporting the conclusions of this article will be made available by the authors, without undue reservation, to any qualified researcher.

## Abbreviations

LPV/r, Lopinavir/ritonavir; 3TC, lamivudine; NRTIs, Nucleoside reverse transcriptase inhibitors; TDF, Tenofovir disoproxil fumarate; EFV, Efavirenz; ART, Antiretroviral therapy; DT, Dual therapy; TT, Triple therapy; ITT, Intention-to-treat; AE, Adverse event; SAE, Severe adverse event; bPI, boosted protease inhibitor; eGFR, estimated glomerular filtration rate; DMSO, dimethyl sulfoxide; PP, per-protocol; SD, standard deviation.
