# Supplementary figures and images for: Antiretroviral Long-Term Efficacy and Resistance of Lopinavir/Ritonavir Plus Lamivudine in HIV-1-Infected Treatment-Naïve Patients (ALTERLL): 144-Week Results of a Randomized, Open-Label, Non-Inferiority Study From Guangdong, China
Source: Front Pharmacol. 2021 Mar 25;11:569766. doi: 10.3389/fphar.2020.569766 (PMC8027496; doi:10.3389/fphar.2020.569766)

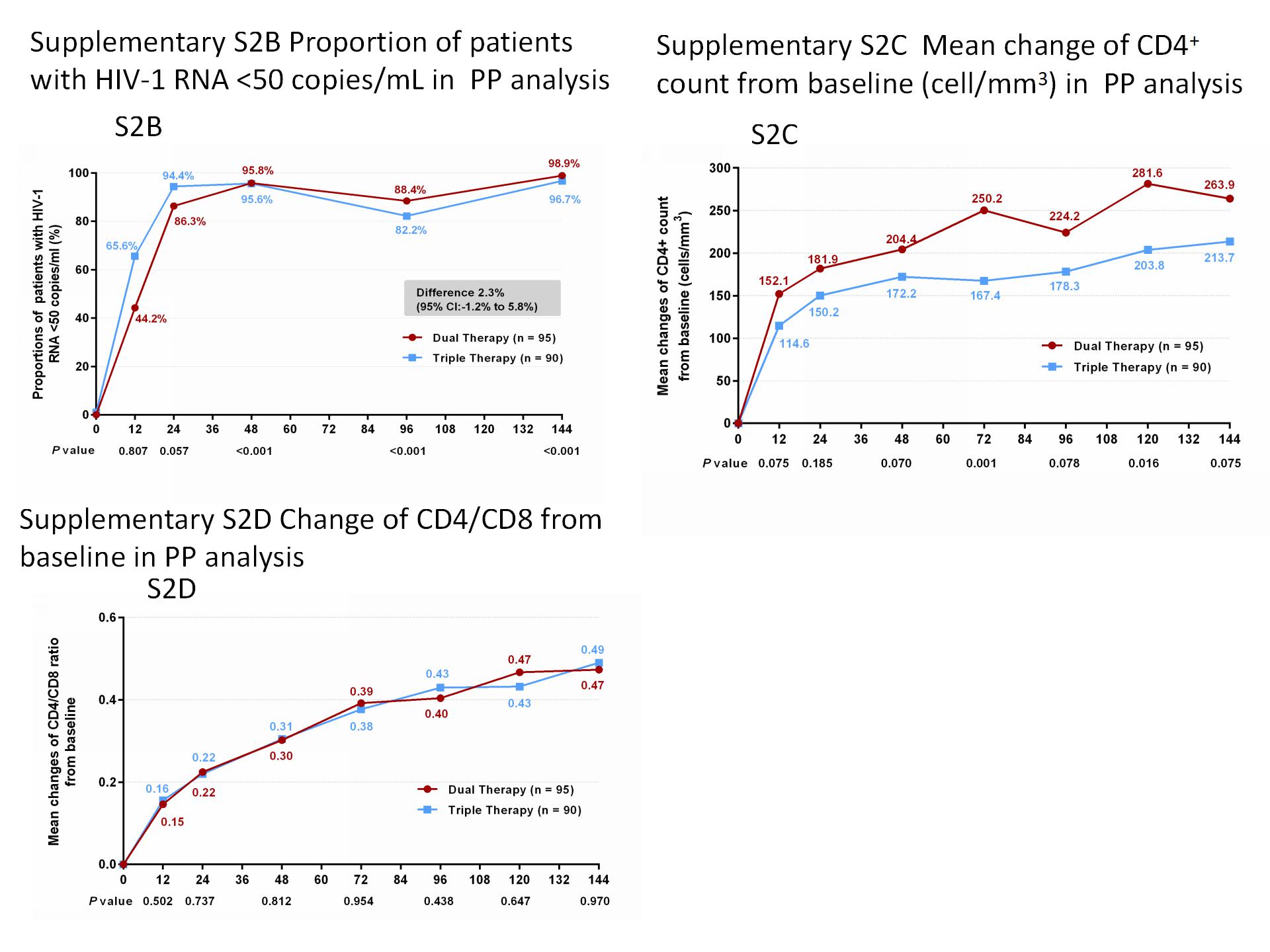

Supplement: Supplementary file 1 [file image1.jpeg]

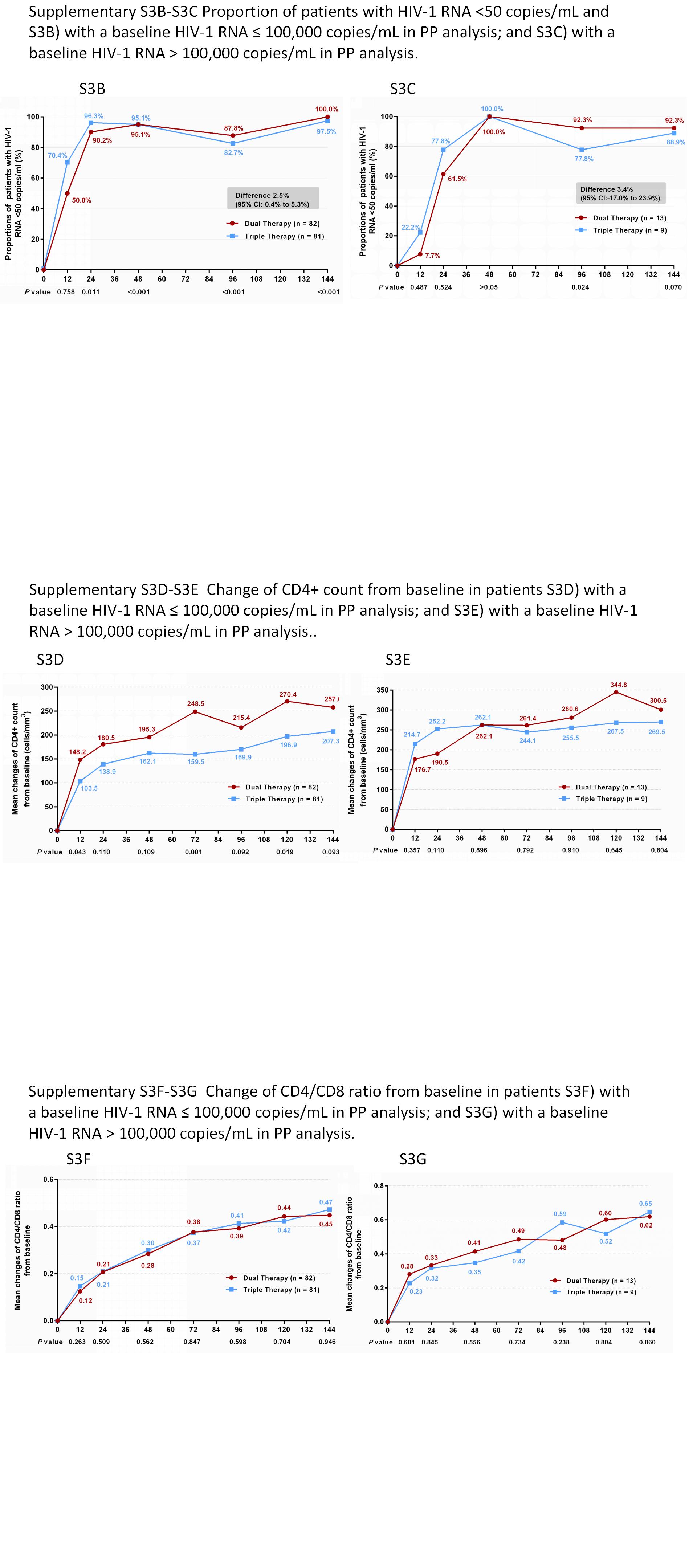

Supplement: Supplementary file 2 [file image2.jpeg]

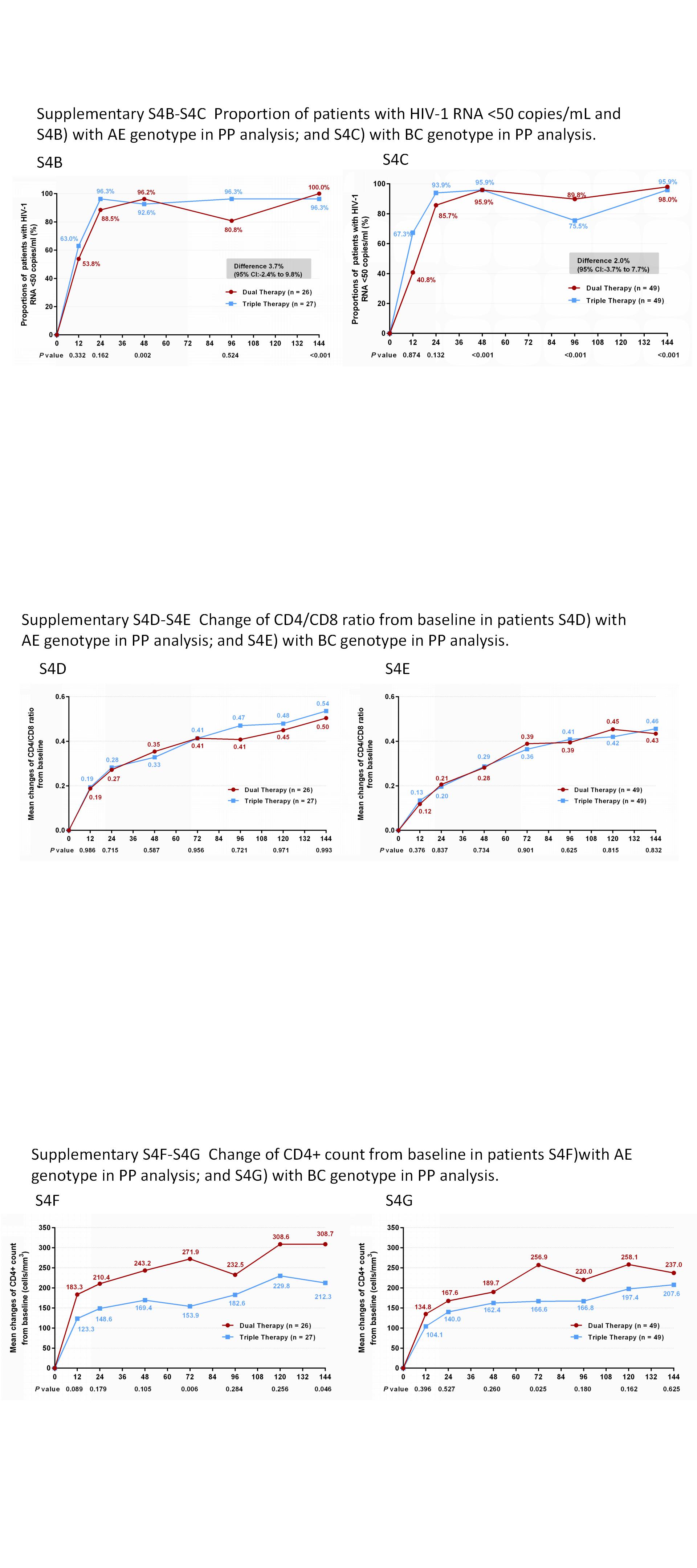

Supplement: Supplementary file 3 [file image3.jpeg]

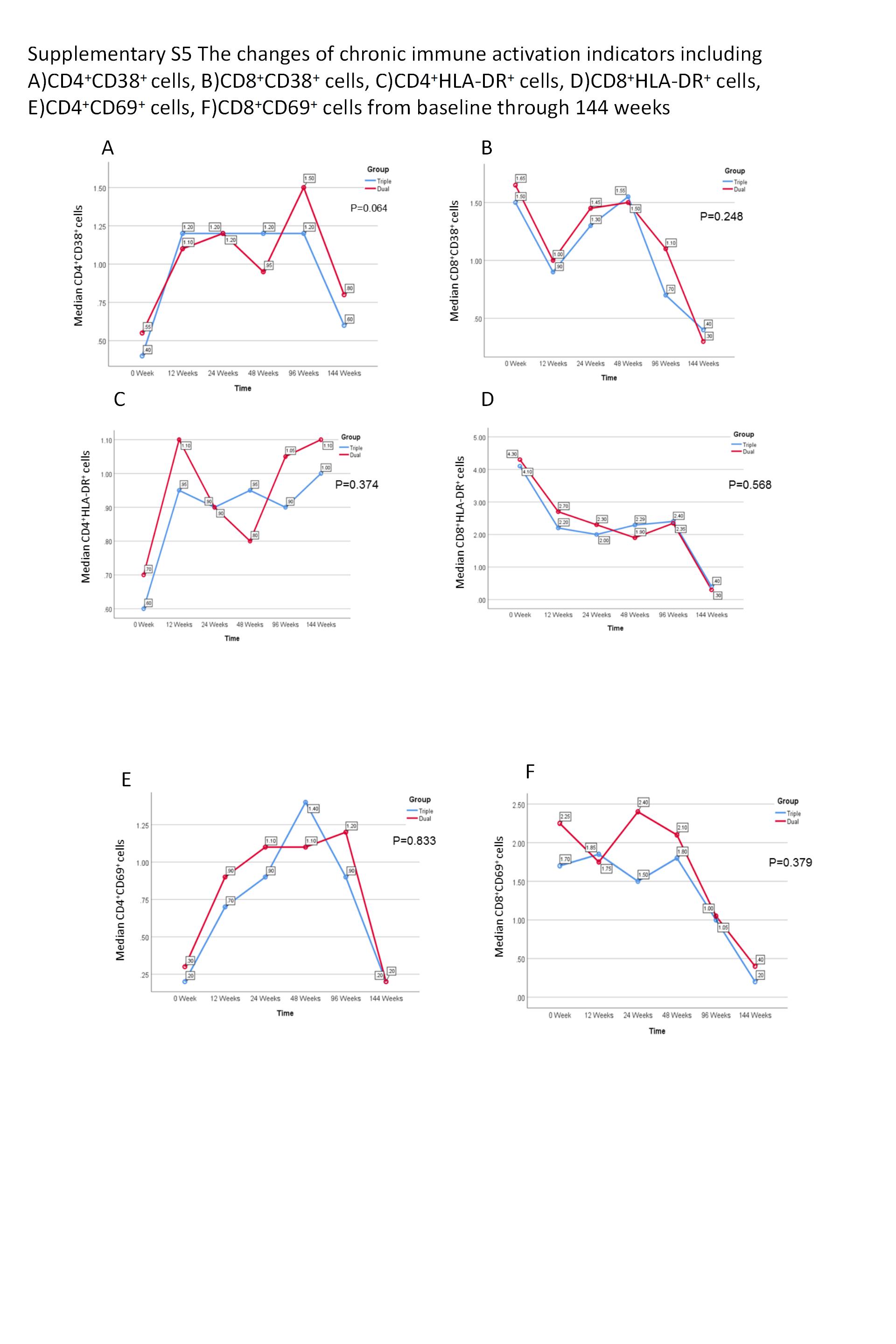

Supplement: Supplementary file 4 [file image4.jpeg]
